# Abundant Extractable Metabolites from Temperate Tree Barks: The Specific Antimicrobial Activity of *Prunus Avium* Extracts

**DOI:** 10.3390/antibiotics9030111

**Published:** 2020-03-04

**Authors:** Amin Abedini, Marius Colin, Jane Hubert, Emilie Charpentier, Apostolis Angelis, Heithem Bounasri, Benjamin Bertaux, Alexis Kotland, Fany Reffuveille, Jean-Marc Nuzillard, Jean-Hugues Renault, Sophie C. Gangloff

**Affiliations:** 1 Biomatériaux et inflammation en site osseux (BIOS) - EA 4691, Université de Reims Champagne Ardenne, 51097 Reims, France; drarjang@gmail.com (A.A.); marius.colin@univ-reims.fr (M.C.); emilie.charpentier@univ-reims.fr (E.C.); fany.reffuveille@univ-reims.fr (F.R.); 2 Institut de Chimie Moléculaire de Reims (ICMR - UMR 7312 CNRS), Université de Reims Champagne Ardenne, 51097 Reims, France; jane.hubert@nat-explore.com (J.H.); aangjel@pharm.uoa.gr (A.A.); jean-marc.nuzillard@univ-reims.fr (J.-M.N.); jh.renault@univ-reims.fr (J.-H.R.); 3 NatExplore SAS, 51430 Prouilly, France; alexis.kotland@nat-explore.com; 4 Department of Pharmacognosy and Natural Products Chemistry, Faculty of Pharmacy, National and Kapodistrian University of Athens, Panepistimiopolis Zografou, 15771 Athens, Greece

**Keywords:** barks, *Prunus avium*, dihydrowogonin, natural products, antimicrobial activity, antibiofilm activity, centrifugal partition chromatography, ^13^C nuclear magnetic resonance chemical profiling

## Abstract

Tree barks are mainly considered as wood wastes from forestry activities, but represent valuable resources as they may contain antimicrobial compounds. Here, we aimed to evaluate the possible antimicrobial activities of bark extracts and to characterize the chemical composition of the most active extract. Ten methanol bark extracts were tested in vitro against 17 bacterial strains and 5 yeast strains, through minimum inhibitory concentration (MIC) and minimum bactericidal (or fungicidal) concentration (MBC/MFC) assays. The extract from *Prunus avium* (E2-4) displayed the largest bactericidal activity against Gram-positive bacteria, with a lethal effect on 6 out of 8 strains. Antibiofilm assays of E2-4 were performed by crystal violet staining and enumeration of adhered bacteria. Assays demonstrated a biofilm inhibitory effect of E2-4 against *Staphylococcus aureus* CIP 53.154 at concentrations equal to or higher than 250 µg/mL. Chemical profiling of E2-4 by ^13^C nuclear magnetic resonance (NMR) revealed the presence of dihydrowogonin as a major constituent of the extract. E2-4 was fractionated by centrifugal partition chromatography and the three fractions containing dihydrowogonin were tested for their antibacterial and antibiofilm activities, revealing similar activities to those of E2-4. Dihydrowogonin was positively assessed as an interesting antimicrobial compound, which could be valued from wastes of *Prunus avium* barks.

## 1. Introduction

The ongoing emergence of resistant bacterial strains has led to the reduced effectiveness of even newly developed antibiotics, making patients much more difficult to treat [1,2,3,4,5]. 

As the number of multidrug-resistant bacteria continues to increase, the number of new antimicrobial molecules continues to dramatically decrease. This phenomenon is becoming a worldwide priority, and indeed a return to a pre-antibiotic era will be a global disaster for modern medicine and human health. Thus, it is urgent to identify low-cost antimicrobial molecules, which prevent the development of bacterial resistance by its action and/or smart use [1,2,3,4,5]. A strategy to find solutions against antibiotic resistance relies on the investigation of natural resources from which highly active compounds may be extracted [6]. The antimicrobial compounds isolated from terrestrial or marine extracts have the ability to inhibit bacterial growth through different mechanisms, as compared to conventional antibiotics from microorganisms, and thus can be of significant clinical value for the treatment of human infectious diseases [7,8]. 

The discovery of new natural antibiotics continues, however, to face important challenges due to the usual complexity of natural extracts. Well known active compounds are for instance frequently rediscovered after laborious bioactivity-guided isolation procedures, or extremely low yields in active substances are reached after purification, limiting further scale-up strategies at pilot or production levels. Additionally, most of the methods employed for the evaluation of the in vitro antimicrobial activity of natural extracts generally provide activity results for crude extracts, but with poor data about sample chemical composition and on potential chemical candidate(s) that could be involved in the observed activity.

Tree barks represent abundant and renewable vegetal resources produced from forestry activities. Barks are most often valued as fuel, insulation materials or simply eliminated as wastes. However, recent studies have highlighted the diversity of biologically active metabolites that can be extracted from barks and upgraded as input materials in high value-added sectors [9,10,11,12]. Bark truly constitutes for every tree its first defense line against external attacks, particularly against infectious agents, and in this sense, is expected to produce compounds, notably polyphenols, potentially as antibacterial drug leads [13].

The aim of the present study was to investigate the potential antibacterial effects of ten bark extracts from ten common deciduous and coniferous tree species growing in temperate forests of North-East France. We tested a collection of bacteria and yeast species involved in many infections and, for which, antibiotic resistance development is increasing. The second objective was to chemically profile the most active extract, and then to test specific fractions of this extract to tentatively identify antibacterial molecules that could be exploited from tree barks.

## 2. Results

### 2.1. Antimicrobial Screening of Ten Methanol Bark Extracts

A large screening of 10 methanol bark extracts (Table 1) was performed by seeding 22 micro-organisms (8 Gram-positive bacteria, 9 Gram-negative bacteria, and 5 yeasts) on agar plate with a multiple inoculator. This technique allowed a first estimation of the minimum inhibitory concentration (MIC) of the bark extracts towards each microorganism to be obtained. The antibiotics used as controls confirmed that all the Gram-negative bacteria were resistant to vancomycin and that all the yeasts were sensitive to amphotericin B. *Enterococcus faecalis* and *Klebsiella pneumoniae* were resistant to both gentamycin and vancomycin. As presented in Table 2, the inhibitory effect on microbial growth, when observed, was obtained within a concentration range of 0.3–10 mg/mL. Gram-positive bacteria were more sensitive to the extracts than the Gram-negative bacteria. One exception, *Shigella sonnei*, was sensitive to all extracts, but at different levels. *Streptococcus pyogenes* growth was disturbed by 8 out of the 10 extracts at concentration ≤0.3 mg/mL. Extracts from *Quercus robur* (E2-2) and *Alnus glutinosa* (E2-3) showed an antimicrobial activity on all the tested microorganisms, with most MICs ≤0.3 mg/mL. *Prunus avium* extract (E2-4) also exhibited an interesting activity profile against all Gram-positive bacteria, all yeasts, and almost all Gram-negative bacteria except *Serratia marcescens* and *Klebsiella pneumoniae*. Extracts from these three species demonstrated the highest antimicrobial activities, followed by *Larix decidua* (E2-8), with an antimicrobial activity observable on only 12 among the 22 tested microorganisms (Table 2).

### 2.2. Microbicidal Activities of the Three Bark Extracts Exhibiting the Lowest MICs

E2-2, E2-3 and E2-4, which exhibited MICs <0.3 mg/mL during the first antimicrobial screening, were further selected to precisely evaluate their antimicrobial potential. Microdilution assays were performed at concentrations ranging from 0.95 µg/mL to 250 µg/mL, to accurately measure MIC and minimum bactericidal (or fungal) concentrations (MBC/MFC). As highlighted in Table 3, the MICs were all comprised between 7.8 and 250 µg/mL. E2-3 showed the lowest MIC (7.8 µg/mL) against *Streptococcus pyogenes*, but none of the three extracts demonstrated MBC against this strain. The MBCs were in general higher than the corresponding MICs and ranged from 31.2 to 250 µg/mL. Overall, the bark extract of *Prunus avium* (E2-4) was the most active in terms of MBC against Gram-positive bacteria, by killing 6 out of 8 Gram-positive bacterial strains at concentrations of 125 µg/mL or 250 µg/mL. This extract also demonstrated MFCs against 3 out of the 5 yeast strains tested. Consequently, E2-4 was considered as the most promising extract and was selected for further antimicrobial tests and chemical characterization.

According to Carbomelle et al. [14], antimicrobial substances can be considered as bactericidal if the ratio MBC/MIC is ≤4 and bacteriostatic agents when the ratio MBC/MIC is >4. Applying these guidelines, 18 MBC/MIC ratios were determined as bactericidal: six for E2-2, three for E2-3, nine for E2-4 (Table 3, yellow boxes). 

### 2.3. Evaluation of Antibiofilm Activity of the Prunus Avium Extract (E2-4)

**Figure 1:**
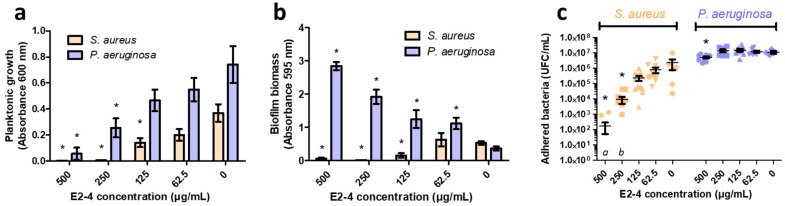
Antibiofilm activity of *Prunus avium* bark extract (E2-4) against *S. aureus* and *P. aeruginosa*, after 24h of contact. (**a**) Evaluation of the planktonic growth of bacteria through optical density measurement at 600 nm. (**b**) Evaluation of the total biofilm biomass through crystal violet coloration and absorbance measurement at 595 nm. (**c**) Concentration of bacteria adhered on coverslips, after ultrasonication and culture on agar plates. * indicates a significant difference (*P* < 0.05) between the condition and the control without E2-4 (0 µg/mL). *a*: 11 replicates did not present any adhered bacteria. *b*: 6 replicates did not present any adhered bacteria.

Antibiofilm assays of E2-4 were performed on *Staphylococcus aureus* (*S. aureus*) CIP 53.154 and *Pseudomonas aeruginosa* (*P. aeruginosa*) ATCC 9027 through analysis of the planktonic growth, coloration of biofilm biomass (matrix and adhered bacteria), by crystal violet and counting of adhered bacteria. E2-4 concentrations ranging from 1000 µg/mL to 62.5 µg/mL were tested in a minimal medium (MM) to favor biofilm formation, leading to different MICs than previously observed in Mueller-Hinton (MH) medium. At 1000 µg/mL, both strains showed no sign of planktonic survival nor biofilm formation (no biomass nor adhered bacteria observed). Between 62.5 µg/mL and 500 µg/mL, the planktonic growth decreased correlatively with the augmentation of E2-4 concentration (Figure 1). The biofilm mass of *S. aureus* was progressively reduced and was correlated to a decreasing number of adhered bacteria in the forming biofilm. On the opposite side, biofilm formation of *P. aeruginosa* was boosted by increasing concentrations of the extract (until 1000 µg/mL), while the number of adhered bacteria remained constant (no significant difference) until 500 µg/mL. 

### 2.4. Chemical Profiling of E2-4 

**Figure 2:**
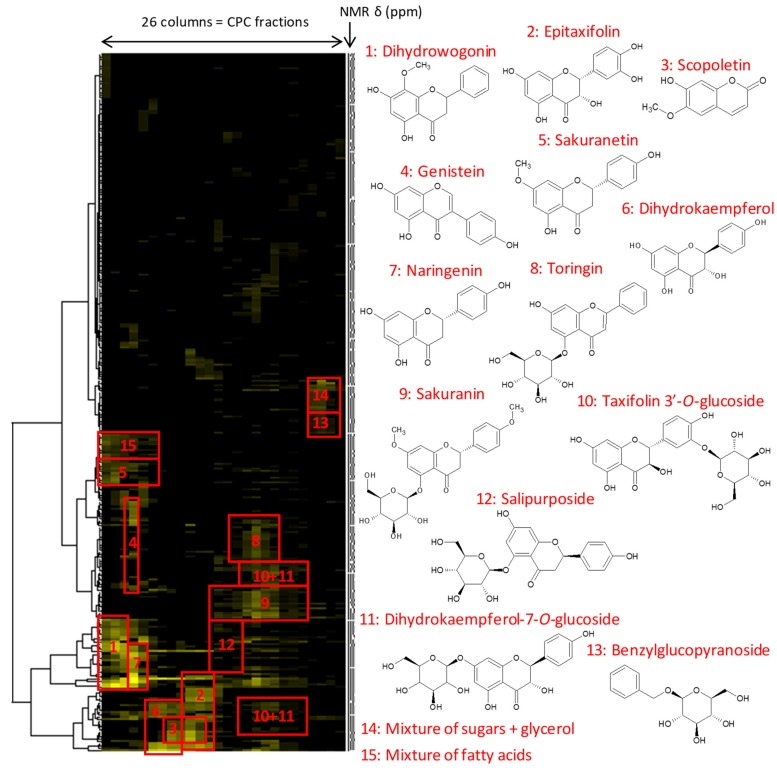
Hierarchical clustering analysis of ^13^C NMR signals detected in all centrifugal partition chromatography (CPC) fractions obtained from the MeOH bark extract of *Prunus avium* (E2-4).

In parallel to these biological assays, the chemical composition of the *Prunus avium* bark extract (E2-4) was investigated by NMR spectroscopy. In order to identify the major constituents, E2-4 was submitted by centrifugal partition chromatography (CPC) to yield 26 fractions, and a Hierarchical Clustering Analysis (HCA) was performed on the ^13^C NMR data of all fractions. The resulting heat map is drawn in Figure 2. The 270 rows of the heat map correspond to the ^13^C chemical shift buckets for which at least one signal was detected in at least one fraction of the extract. The higher the intensity of ^13^C NMR peaks, the brighter the yellow color in the map. The map highlights strongly correlated ^13^C NMR chemical shifts, belonging to the major chemical structures present in E2-4. These clusters of NMR chemical shifts were used one by one as keys to mine an in-house database containing the structures and predicted ^1^H and ^13^C NMR chemical shifts of a range of natural products (n ≈ 4000 in January 2020), allowing the identification of all the constituents presented in Figure 2. The confidence level of ^13^C chemical shift cluster annotation was increased by a complementary analysis of 2D HSQC, HMBC and COSY NMR spectra, performed on CPC fractions. A high diversity of flavonoids was identified, mainly derived from flavanones and flavonols in their aglycone form. Dihydrowogonin was identified as the major constituent, followed by (+)-epitaxifolin and by the coumarin scopoletin, which were both present in significant amounts. Other glycosylated flavonoids derived from kaempferol, taxifolin and naringenin were also detected in the most polar fractions of the extract, but in lower levels.

As dihydrowogonin was the major chemical marker of E2-4, and a potentially strong antimicrobial compound due to its phenolic chemical structure, further works will focus on the fractions containing this compound.

### 2.5. Antimicrobial and Antibiofilm Activity of Three Fractions from E2-4

**Figure 3:**
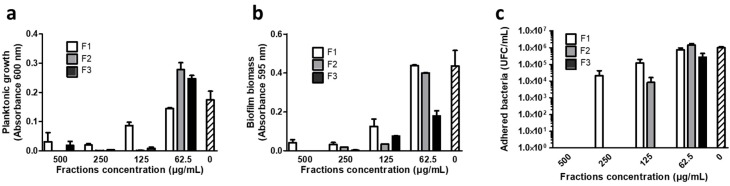
Antibacterial and antibiofilm activity E2-4 fractions containing dihydrowogonin as the major compound (F1, F2 and F3) against *S. aureus* after 24 h of contact. Hatched bars: no fraction. (**a**) Evaluation of the planktonic growth of bacteria through optical density measurement at 600 nm. (**b**) Evaluation of the total biofilm biomass through crystal violet coloration and absorbance measurement at 595 nm. (**c**) Concentration of bacteria adhered on coverslips, after ultrasonication and culture on agar plates.

Antimicrobial and antibiofilm assays of fractions enriched in dihydrowogonin (F1, F2 and F3) were performed with the same protocols as described in Section 2.3, on *S. aureus* CIP 53.154. Results are presented in Figure 3. The three fractions showed a similar effect as observed for the whole extract E2-4, with a strong decrease in both planktonic growth and biofilm formation for fraction concentrations of 125 µg/mL and above. This effect was particularly important for fractions 2 and 3, while the decrease was more progressive for F1, with more residual bacteria and biofilm formation at high concentrations.

## 3. Discussion

Over the last years, an increasing number of studies have brought to the fore the diversity of biologically active extracts or metabolites that can be obtained from temperate trees [12,13,15,16,17]. In a difficult context marked by the widespread increase of microorganism resistance and, therefore, by an urgent need of new antibiotics, it may be assumed that tree barks represent a relevant source of candidates.

In the present study, ten methanol bark extracts obtained from temperate trees were tested simultaneously against a panel of 22 microorganisms for their growth inhibitory and microbiocidal activities. A preliminary chemical profiling of these 10 bark extracts was previously reported [17] and it represented a crucial first step to identify potentially valuable molecules. This previous study also approached the antibacterial activity of these bark extracts, but through a non-quantitative method and against one bacterial strain only (*S. aureus*). Here, we focused on the accurate antibacterial activity by determining both MIC and MBC, and further through the antibiofilm activity.

Although some extracts exhibited a wide spectrum of activity against all the tested microorganisms, Gram-positive bacteria and yeasts were overall more sensitive than the Gram-negative bacteria. This can be due to different parameters, including density and/or localization of the targets, molecular diffusion, influx and efflux mechanisms, and of course to the chemical structures of the different constituents of the extracts. For example, most of the tested bark extracts were active against *Streptococcus pyogenes*, with quite low MICs (E2-3 MIC = 7.8 µg/mL, E2-2 and E2-4 MIC = 31.2 µg/mL). Even if no MBC could be determined for this strain, this result is very interesting as *S. pyogenes* is a Gram-positive bacterium which causes more than 600 million infections per year, colonizing the upper respiratory tract and skin of asymptomatic individuals [18,19]. Even though *S. pyogenes* has remained universally susceptible to β-lactams and glycopeptides, the rate of antibiotic failure against this bacterium has dramatically increased over the past 15 years, reaching up to 40% in some regions of the world [20]. Therefore, being able to add some alternative molecules in the therapeutic arsenal will be of great interest.

Based on the overall results of MIC and MBC determination, three tree species stood out with interesting antimicrobial activities: *Quercus robur*, *Alnus glutinosa* and *Prunus avium*, respectively E2-2, E2-3 and E2-4. Only the methanol bark extract of *Prunus avium* (E2-4) was bactericidal against a panel of strains (Table 3, nine boxes). The global objective of this study was to identify the antimicrobial activity of major compounds of the extracts, to be further extracted at a larger scale.

The antibacterial activity, fractionation and chemical characterization of E2-3 has previously been described [12]. The study demonstrated that oregonin was predominant in the extract, and fractions containing oregonin as major compound exerted the highest antimicrobial activities. *Quercus robur* (E2-2) is already known to produce valuable polyphenols, like ellagic acid, which display strong antibacterial activities, suggesting a promising clinical potential to treat human infectious diseases [13,21,22,23]. Here, we focused on the *Prunus avium* extract (E2-4), as it presented good antimicrobial activities and especially because it was, by far, the best bactericide against Gram-positive bacteria.

The chemical profiling of E2-4 enabled correlation of the antimicrobial effects to the presence of particular chemical constituents and thus, the determination of the metabolites involved in the observed activities. Dihydrowogonin was identified as an abundant metabolite of the methanol extract of *Prunus avium* bark. The fractions enriched in dihydrowogonin, especially F2 and F3, showed slightly higher antibacterial and antibiofilm activities than extract E2-4 against *S. aureus* (Figure 1, Figure 2 and Figure 3). Comparing MIC and MBC of ellagic acid [13] and dihydrowogonin against *S. aureus*, they both appeared to be lower for dihydrowogonin, suggesting that it may be considered as an effective compound against Gram-positive bacteria, while it is less active against Gram-negative bacteria, especially against *P. aeruginosa*. This article is the first report of the antimicrobial activity of dihydrowogonin, to the best of our knowledge. Interestingly, dihydrowogonin has already been detected in leaves extracts of *P. avium*, and seems to play a role in *syrB* gene activation in *Pseudomonas syringae*, a known phytopathogen [24].

Biofilm construction also constitutes an efficient strategy to drastically increase tolerance against antimicrobial agents [25]. Thus, it is of the greatest importance to find new compounds with inhibitory effects on biofilm formation. Biofilm formation could be promoted by antibiotics under or at MIC, as bacterial defensive strategy [26,27]. In this study, E2-4 and its fractions proved to progressively inhibit biofilm formation of *S. aureus*, even at low concentrations. The biofilm reduction seems to be linked to both a reduction of the matrix production and a decrease in the number of adhered bacteria when the extract or the dihydrowogonin-enriched fractions are present. Conversely, *P. aeruginosa* produced more biofilm matrix in the presence of increasing concentrations of E2-4 extract. This bacterium is well known for its ability to produce robust biofilm, especially under stressful conditions. We speculate that the production of matrix increases under the treatment stress as a protection defense until the concentration of 1000 µg/mL, which totally inhibited planktonic growth and biofilm development.

However, this in vitro approach on antibiofilm activity represents preliminary data, necessary to identify a molecule of interest. Further investigations are needed, particularly on specific biofilm models.

The next step would be to precisely evaluate the mechanisms of action of pure dihydrowogonin against bacterial strains or yeasts, to finally determine the potential use of this molecule as an interesting antimicrobial metabolite. Its chemical stability, due to the presence of an unsubstituted B-ring and methoxylated group [28], makes it a potentially interesting molecule to be used as an antiseptic or a disinfectant. 

## 4. Materials and Methods

### 4.1. Chemicals, Reagents, and Bark Materials

Methanol (MeOH) and *n-*heptane were purchased from Carlo Erba Reactifs SDS (Val de Reuil, France). Deuterated methanol (methanol-*d4*) was purchased from Sigma-Aldrich (Saint-Quentin-Fallavier, France). An identification number was assigned to each of the investigated species: the common beech *Fagus sylvatica* (1), the pedunculate oak *Quercus robur* (2), the black alder *Alnus glutinosa* (3), the wild cherry *Prunus avium* (4), the sycamore maple *Acer pseudoplatanus* (5), the ash *Fraxinus excelsior* (6), the poplar *Populus x canadensis* (Robusta) (7), the European larch *Larix decidua* (8), the Norway spruce *Picea abies* (9), and the Eurasian aspen *Populus tremula* (10). The MeOH bark extracts of each tree species were prepared as previously reported [17]. Barks were collected in the forest of Signy l’Abbaye (Grand Est region, France), in October 2014. After drying at 30 °C for 72 h and grinding by a hammer mill, two successive solid-liquid extractions with 3 L of *n-*heptane (E1) and then 3 L of MeOH (E2) were performed on 200 g of the powdered barks under magnetic stirring for 24 h at room temperature. The resulting solutions were filtered, and the solvents were evaporated under reduced pressure. As a result, 20 extracts were obtained from E1-1 to E1-10 (n-heptane extracts) and from E2-1 to E2-10 (MeOH extracts).

### 4.2. Microorganisms and Culture Media

Among the 22 strains used in this study, 5 were yeast strains including *Candida glabrata* (clinical strain, lab. collection), *Candida tropicalis* (clinical strain, lab. collection), *Candida kefyr* (clinical strain, lab. collection), *Candida albicans* (clinical strain, lab. collection), and *Cryptococcus neoformans* (clinical strain, lab. collection), and 17 were bacterial strains with 8 Gram-positive bacteria, *Bacillus subtilis* ATCC 6633, *Enterococcus faecalis* ATCC 1034, *Staphylococcus aureus* NCTC 8325, *Staphylococcus aureus* CIP 53.154, *Staphylococcus epidermidis* (clinical strain, lab. collection), *Streptococcus pyogenes* (clinical strain, lab. collection), *Micrococcus luteus* (clinical strain, lab. collection), *Listeria innocua* (clinical strain, lab. collection) and 9 Gram-negative bacteria, *Providencia stuartii* (clinical strain, lab. collection), *Serratia marcescens* (clinical strain, lab. collection), *Proteus vulgaris* (clinical strain, lab. collection), *Klebsiella pneumoniae* (clinical strain, lab. collection), *Pseudomonas aeruginosa* ATCC 9027, *Shigella sonnei* (clinical strain, lab. collection), *Escherichia coli* CIP 54.127, *Enterobacter cloacae* (clinical strain, lab. collection), and *Salmonella enterica* (clinical strain, lab. collection). All strains were cultured on Müller-Hinton agar (MHA) and in Müller-Hinton broth (MH) (Biokar, Beauvais, France).

### 4.3. Antimicrobial Screening of the Ten Methanol Bark Extracts

The in vitro antibacterial activity of all *n-*heptane extracts and all methanol extracts was previously screened by bio-autography against *Staphylococcus aureus* CIP 53.154 strain, revealing that bacterial growth was not significantly inhibited by the presence of the n-heptane extracts, while all methanol extracts displayed an antibacterial activity [17]. Therefore, the work presented here was only performed on the ten methanol bark extracts, referenced E2-1 to E2-10. Extracts were solubilized in methanol and then diluted in the bacteria culture medium. We controlled the impact of the percentage of methanol in our controls.

Experiments were performed according to internationally recognized guidelines [29]. The 22 microbial strains were firstly stripped aerobically on Mueller-Hinton (MH) agar, then incubated overnight at 37°C in tubes containing the MH broth medium, and finally diluted with MH broth by means of serial dilution, to finally reach a concentration of 10^5^ bacteria/mL.

The growth inhibitory concentrations of the methanol bark extracts were first determined using the agar dilution method [12]. Briefly, the 22 microorganisms were seeded by a multiple inoculator on agar plates containing decreasing concentrations of the extracts (10, 5, 2.5, 1.2, 0.6 and 0.3 mg/mL). After incubation at 37 °C for 24 h, the presence or absence of colonies was detected by visual inspection. The minimum inhibitory concentration (MIC) values were recorded as the lowest concentrations of compounds, for which no growth of the microorganisms was observed. Methanol was also checked in the same concentration as in the samples for absence of antibacterial activity. Three antibiotics including gentamicin (G), vancomycin (V) and amphotericin B (A) were used as growth inhibitor positive controls.

For all methanol extracts exhibiting MIC on agar plate at a concentration of less than 0.3 mg/mL, the MH broth microdilution method was used in order to determine more precisely their MIC against the selected bacteria [30]. For this purpose, nine decreasing concentrations of extracts (two-fold dilution from 250 µg/mL to 0.95 µg/mL) were used in 96-well microtiter plates, against a 10^5^ bacteria/mL suspension in a final volume of 200 µL. The bacteria culture control (no extract added) and medium sterility control (no inoculum added) were included. The plates were incubated overnight at 37 °C. Bacterial growth was evaluated both visually and after spraying 0.2 mg/mL iodonitrotetrazolium chloride (INT, Sigma, France) to each well. After incubation at 37 °C for 30 min, bacterial growth was determined by a reddish-pink color. MIC values were determined as the lowest concentrations of extracts having an inhibitory effect on bacterial growth (clear wells). This test was performed in duplicate. The minimum bactericidal concentrations (MBC) and minimum fungicidal concentrations (MFC) were also determined after subculture of 100 µL of the well samples with no visible bacterial growth before the INT spraying step. After 24 h of incubation, the lowest concentration of the subculture showing no growth was considered as the MBC or MFC.

### 4.4. Static Biofilm Assays

*S. aureus* CIP 53.154 and *P. aeruginosa* ATCC 9027 strains were cultivated overnight in nutrient medium. Then, a minimal medium (MM) was used to favor biofilm formation. MM is composed of 62 mM potassium phosphate buffer at pH 7.0; 7 mM (NH4)2SO_4_; 2 mM MgSO_4_; 10 μM FeSO_4_; 0.4% (*w*/*v*) of glucose and 0.5% (*w*/*v*) of casamino acids. Overnight cultures were diluted to the hundredth in MM and 500 µL was distributed in each well of a 24-well microtiter plate. After 24 h of incubation at 37 °C, the planktonic growth was evaluated by measuring the absorbance at 600 nm (A600; results are expressed with the subtraction of the blank: medium without bacteria). Then, the plates were gently washed three times with PBS and biofilm biomass was evaluated by adding 0.2% of crystal violet stain for 20 min [26]. After washing, 500 µL of 95% ethanol was added to each well. The absorbance at 595 nm was measured to quantify the amount of biofilm (results are expressed with the subtraction of the blank: medium without bacteria). In addition, a plastic coverslip (ThermanoxTM, Nunc, Denmark) was present at the bottom of each well to quantity live adhered bacteria. After 24 h of incubation, the coverslip was washed in MM and bacteria were then detached by exposing the sample to 5 min of ultrasound (40 kHz). Serial dilutions were plated on nutrient agar plates to evaluate the quantity of attached bacteria.

### 4.5. Centrifugal Partition Chromatography (CPC)

The *Prunus avium* MeOH bark extract was fractionated by CPC on a lab-scale FCPE300® column of 303 mL capacity (Rousselet Robatel Kromaton, Annonay, France), as described in the publication of Abedini et al. [12] Briefly, 2.8 L of the three-phase solvent system *n-*heptane/ MtBE/CH_3_CN/water (1/1/1/1, *v*/*v*) were prepared in a separatory funnel. The upper phase (UP) was set aside and 700 mL of MtBE were further added to the remaining mixture of the medium phase (MP) and lower phase (LP). The lower phase was introduced into the column as the stationary phase. The rotation speed was then set at 1200 rpm. The sample was solubilized in a mixture of LP/MP/UP in the proportions (45/10/5, *v*/*v*) and loaded into the column. The UP was used as the first mobile phase at 20 mL/min for 50 min in order to elute the less polar compounds. Then, the MP was used as the second mobile phase for 60 min to obtain the compounds of medium polarity. Finally, the most polar compounds were obtained by extrusion of the stationary phase for 30 min. Fractions were collected every minute and combined according to their thin layer chromatography profile similarities (data not shown), resulting in a final series of 26 fractions.

### 4.6. NMR Analyses, Principal Component Analysis and Dereplication of the Major Potentially Active Compounds

Aliquots (≈ 20 mg) of all CPC fractions (*n* = 26) obtained from E2-4 were dissolved in 600 µL methanol-*d4* and analyzed by ^13^C NMR spectroscopy at 298 K on a Bruker Avance AVIII-600 spectrometer (Karlsruhe, Germany) equipped with a TXI cryoprobe. ^13^C NMR spectra were acquired at 150.91 MHz using a standard zgpg pulse sequence, with an acquisition time of 0.9 s, a relaxation delay of 3 s, and a total of 1024 scans. After spectra processing using the TOPSPIN 3.5 software (Bruker), the absolute intensities of all ^13^C NMR signals detected in all spectra were collected by automatic peak picking. Then, the ^13^C NMR spectral width (from 0 to 240 ppm) was divided into chemical shift buckets of 0.2 ppm and the absolute intensity of the NMR peaks detected in all spectra was associated to the corresponding bucket. This step was performed using a locally developed computer script written in the Python language, resulting in a table with 26 columns corresponding to the CPC fractions, and 270 rows corresponding to the NMR spectral buckets, for which at least one ^13^C NMR peak was detected in at least one spectrum. A hierarchical clustering analysis (HCA) was performed on the rows for the data visualization of signals corresponding to major compounds contained in the E2-4 extract. The proximity between samples was measured with the Euclidian distance and data agglomeration was performed with the Ward’s method. The resulting clusters of ^13^C NMR chemical shifts were visualized as dendrograms on a heat map (Figure 2). 

In parallel, a literature survey was performed to find the names and molecular structures of metabolites already described in the literature for *Prunus avium.* Their ^13^C NMR spectra were predicted by means of the ACD/NMR Workbook Suite 2012 software (ACD/Labs, Toronto, ON, Canada) and stored in a local database already containing the structures and predicted chemical shifts of around 4000 natural metabolites (January 2020). The ^13^C NMR chemical shifts regrouped with the HCA were submitted to the database in order to identify the corresponding chemical structures. 

### 4.7. Statiscal Methods

Statistical comparisons of a series of quantitative values were performed by the non-parametric Mann–Whitney test, using Prism software (version 5, GraphPad Software, San Diego, CA, USA). The results were considered statistically significant when *P* ≤ 0.05.

## 5. Conclusions

Ten methanol bark extracts obtained from common temperate tree species were simultaneously evaluated for their antimicrobial activities against a panel of 22 microorganisms. As a first result, three methanol bark extracts were identified as promising sources of microbicidal agents: *Quercus robur* (E2-2), *Alnus glutinosa* (E2-3) and *Prunus avium* (E2-4) with activity against Gram-positive, Gram-negative bacteria and yeast. The high antimicrobial and antibiofilm activity of *Prunus avium* bark extract is undoubtedly linked to the presence of dihydrowogonin, and is thus revealed as an original and promising candidate antimicrobial compound. Further studies will be carried out to isolate this compound in pure state, in order to quantify at best its antimicrobial activity and to clearly understand the mechanisms involved in its bacteriostatic and bactericidal actions.

## Figures and Tables

**Table 1:** Names of tree species and associated code of bark extracts.

Extract Code	Binomial Name	Common Name
E2-1	*Fagus sylvatica*	Common beech
E2-2	*Quercus robur*	Pedunculate oak
E2-3	*Alnus glutinosa*	Black alder
E2-4	*Prunus avium*	Wild cherry
E2-5	*Acer pseudoplatanus*	Sycamore maple
E2-6	*Fraxinus excelsior*	European ash
E2-7	*Populus x canadensis* (Robusta)	Canadian poplar
E2-8	*Larix decidua*	European larch
E2-9	*Picea abies*	Norway spruce
E2-10	*Populus tremula*	Eurasian aspen

**Table 2:** Determination on agar plates of the minimum inhibitory concentration (MIC) values of the ten methanol bark extracts against 22 microbial species. Tested concentrations ranged from 0.3 mg/mL to 10 mg/mL.

Micro-Organism		MIC (mg/mL)										Positive Controls			
		E2-1	E2-2	E2-3	E2-4	E2-5	E2-6	E2-7	E2-8	E2-9	E2-10	G		V	A
Gram positive bacteria	*Bacillus subtilis*	5	≤0.3	≤0.3	≤0.3	5	1.2	2.5	1.2	1.2	2.5	S		S	NT
	*Enterococcus faecalis* ATCC 1034	10	≤0.3	≤0.3	≤0.3	NA	5	5	1.2	2.5	5	R		R	NT
	*Staphylococcus aureus* NCTC 8325	0.6	≤0.3	≤0.3	≤0.3	NA	2.5	2.5	≤0.3	1.2	5	S		S	NT
	*Staphylococcus aureus* CIP 53.154	≤0.3	≤0.3	≤0.3	≤0.3	NA	1.2	2.5	1.2	2.5	2.5	S		S	NT
	*Staphylococcus epidermidis*	0.6	≤0.3	≤0.3	≤0.3	10	1.2	2.5	≤0.3	≤0.3	1.2	S		S	NT
	*Listeria innocua*	5	≤0.3	≤0.3	≤0.3	5	2.5	5	1.2	1.2	2.5	S		S	NT
	*Streptococcus pyogenes*	5	≤0.3	≤0.3	≤0.3	≤0.3	≤0.3	2.5	≤0.3	≤0.3	≤0.3	S		S	NT
	*Micrococcus luteus*	1.2	≤0.3	≤0.3	≤0.3	5	2.5	2.5	≤0.3	≤0.3	2.5	S		S	NT
Gram negative bacteria	*Escherichia coli* CIP 54.127	NA	2.5	0.6	10	NA	NA	NA	NA	NA	NA	S		R	NT
	*Enterobacter cloacae*	NA	2.5	0.6	10	NA	NA	NA	NA	NA	NA	S		R	NT
	*Salmonella enterica*	NA	1.2	0.6	10	NA	NA	NA	NA	NA	NA	S		R	NT
	*Serratia marcescens*	NA	1.2	≤0.3	NA	NA	NA	NA	NA	NA	NA	S		R	NT
	*Proteus vulgaris*	NA	≤0.3	≤0.3	2.5	NA	NA	NA	NA	NA	NA	S		R	NT
	*Klebsiella pneumoniae*	NA	2.5	0.6	NA	NA	NA	NA	NA	NA	NA	R		R	NT
	*Providencia stuartii*	NA	≤0.3	≤0.3	2.5	NA	NA	NA	NA	NA	NA	S		R	NT
	*Pseudomonas aeruginosa* ATCC 9027	NA	≤0.3	≤0.3	10	NA	NA	NA	NA	NA	NA	S		R	NT
	*Shigella sonnei*	0.6	≤0.3	≤0.3	≤0.3	10	1.2	2.5	≤0.3	0.6	1.2	S		I	NT
Yeast	*Candida glabrata*	NA	≤0.3	≤0.3	≤0.3	NA	NA	NA	10	NA	NA	R		R	S
	*Candida tropicalis*	NA	≤0.3	0.6	≤0.3	NA	NA	NA	≤0.3	NA	NA	R		R	S
	*Candida kefyr*	NA	≤0.3	≤0.3	≤0.3	NA	NA	NA	NA	NA	NA	R		R	S
	*Candida albicans*	NA	≤0.3	0.6	≤0.3	NA	NA	NA	≤0.3	NA	NA	R		R	S
	*Cryptococcus neoformans*	2.5	≤0.3	≤0.3	≤0.3	2.5	NA	NA	NA	NA	NA	R		R	S

NT: Non tested; NA: No activity; S: Sensitive; I: Intermediate; R: Resistant. MIC (µg/mL) of positive controls: Gentamicin (G), S: ≤4, R: >8; Vancomycin (V), S: ≤4, R: >16; Amphotericin B (A), S: ≤1, R: >4. Green boxes: MIC <0.3 mg/mL; Yellow boxes: 0.3 mg/mL< MIC <10 mg/mL; Red boxes: 10 mg/mL< MIC.

**Table 3:** MIC, minimum bactericidal (or fungicidal) concentration (MBC/MFC) values for the three best antimicrobial extracts, determined by microdilution assays. Tested concentrations ranged from 7.8 µg/mL to 250 µg/mL.

Micro-Organism		µg/mL					
		E2-2		E2-3		E2-4	
		MIC	MBC	MIC	MBC	MIC	MBC
Gram positive bacteria	*Bacillus subtilis*	62.5	NA	125	NA	62.5	125
	*Enterococcus faecalis* ATCC 1034	62.5	NA	125	NA	62.5	250
	*Staphylococcus aureus* NCTC 8325	62.5	250	62.5	250	62.5	125
	*Staphylococcus aureus* CIP 53.154	31.2	NA	62.5	NA	62.5	125
	*Staphylococcus epidermidis*	31.2	NA	62.5	NA	62.5	NA
	*Listeria innocua*	31.2	125	125	250	62.5	125
	*Streptococcus pyogenes*	31.2	NA	7.8	NA	31.2	NA
	*Micrococcus luteus*	31.2	62.5	62.5	NA	62.5	250
Gram negative bacteria	*Serratia marcescens*	-	-	62.5	NA	-	-
	*Proteus vulgaris*	62.5	NA	31.2	NA	-	-
	*Providencia stuartii*	15.6	NA	125	NA	-	-
	*Pseudomonas aeruginosa* ATCC 9027	31.2	NA	125	125	-	-
	*Shigella sonnei*	15.6	NA	250	NA	125	NA
		**MIC**	**MFC**	**MIC**	**MFC**	**MIC**	**MFC**
Yeast	*Candida glabrata*	31.2	125	125	NA	125	NA
	*Candida tropicalis*	31.2	NA	-	-	31.2	250
	*Candida kefyr*	62.5	250	125	NA	62.5	250
	*Candida albicans*	62.5	125	-	-	125	125
	*Cryptococcus neoformans*	125	NA	125	NA	62.5	NA

NT: Non tested; NA: No activity. Green boxes: MIC <100 µg/mL; Red boxes: 100 µg/mL< MIC; Yellow boxes: MBC of MFC detected.
